# Supplementary material for: Content of Phenolic Acids in the Grain of Selected Polish Triticale Cultivars and Its Products
Source: Molecules. 2021 Jan 21;26(3):562. doi: 10.3390/molecules26030562 (PMC7865965; doi:10.3390/molecules26030562)
Supplement: Supplementary file 1 [file molecules-26-00562-s001.zip › Supplementary II.pdf]

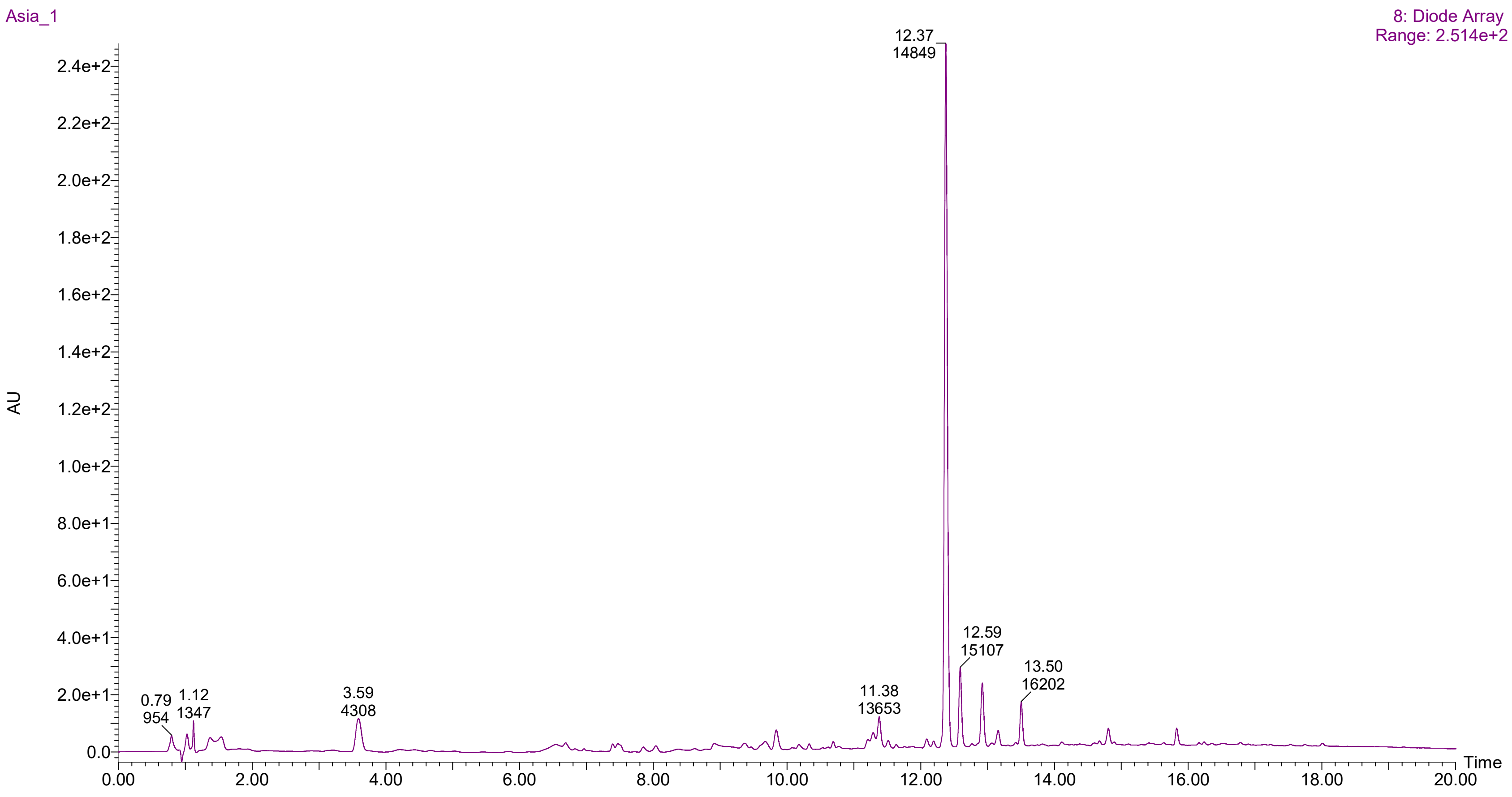

Figure 1. UPLC chromatogram of triticale grain variety Borwo

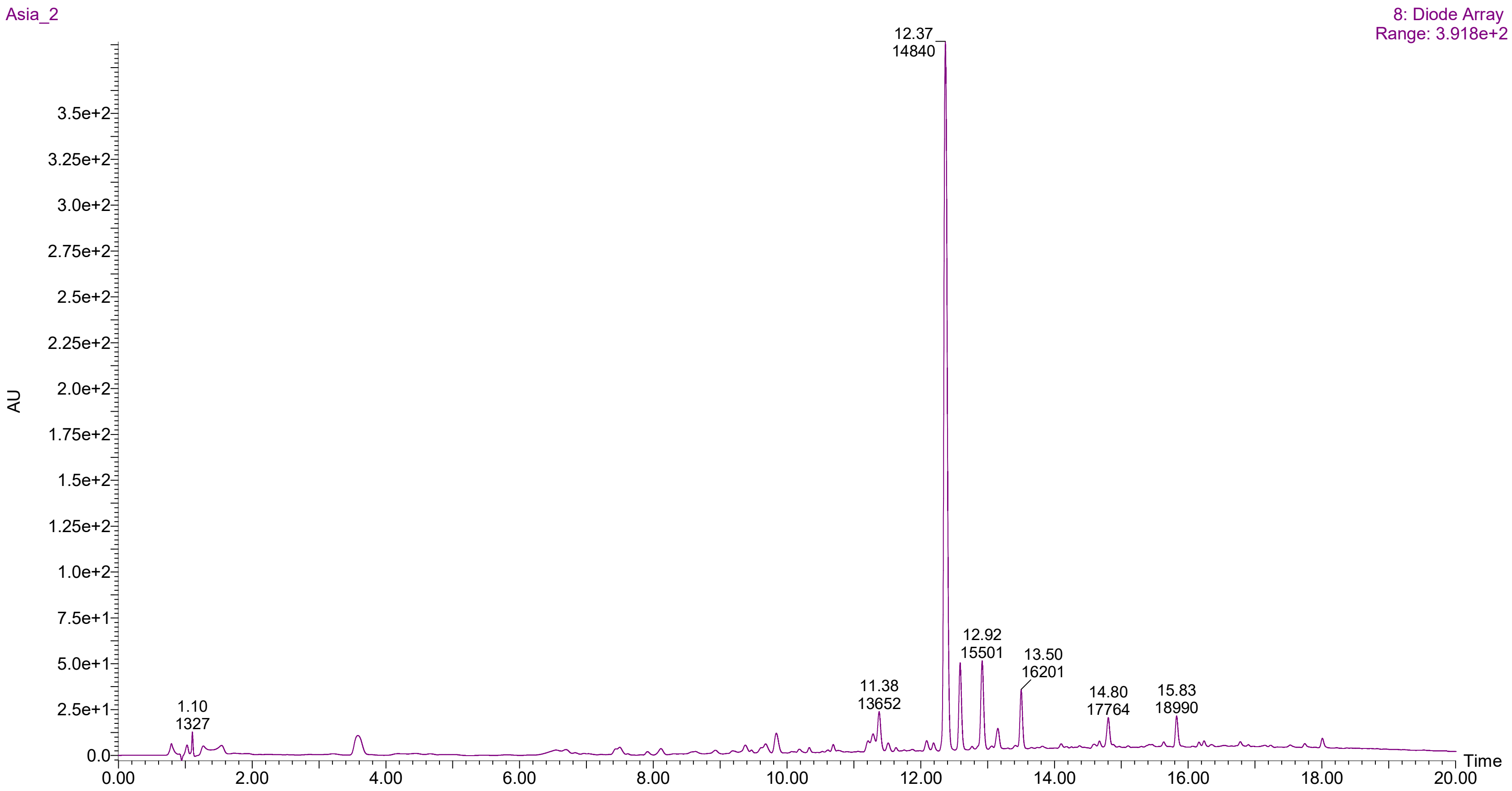

Figure 2. UPLC chromatogram of triticale grain variety Fredro

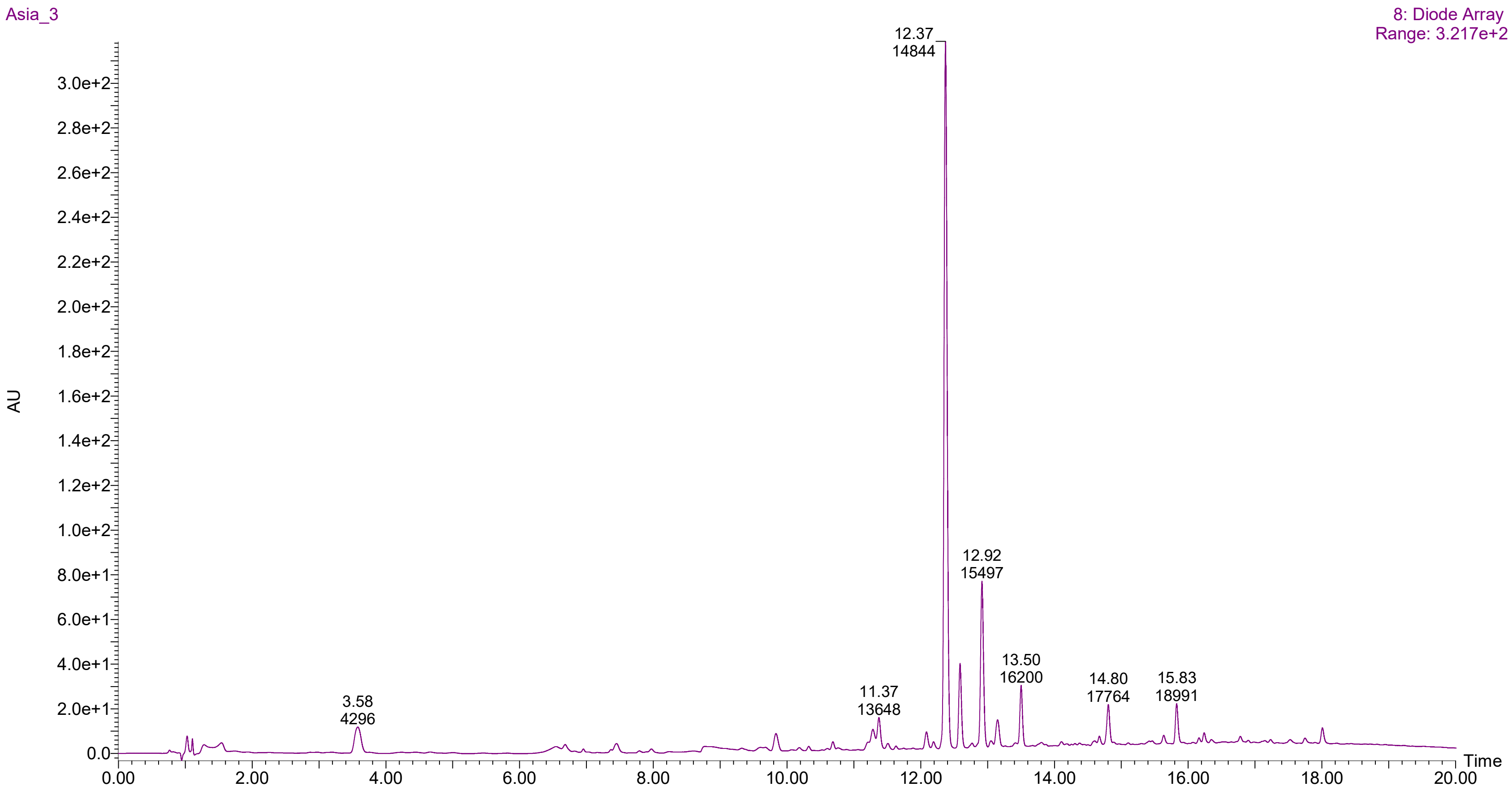

Figure 3. UPLC chromatogram of triticale grain variety Pantenon

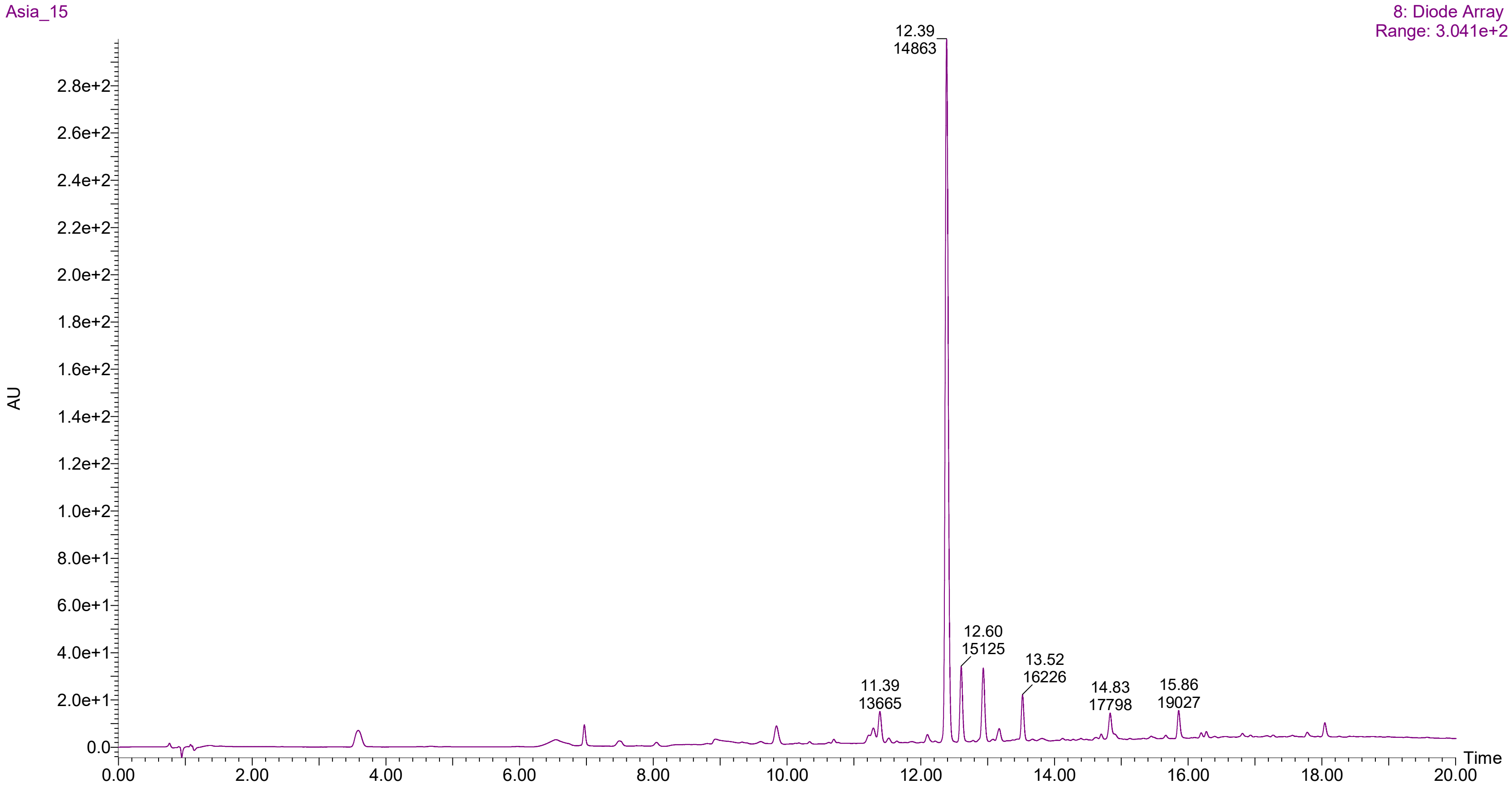

Figure 4. UPLC chromatogram of triticale flour

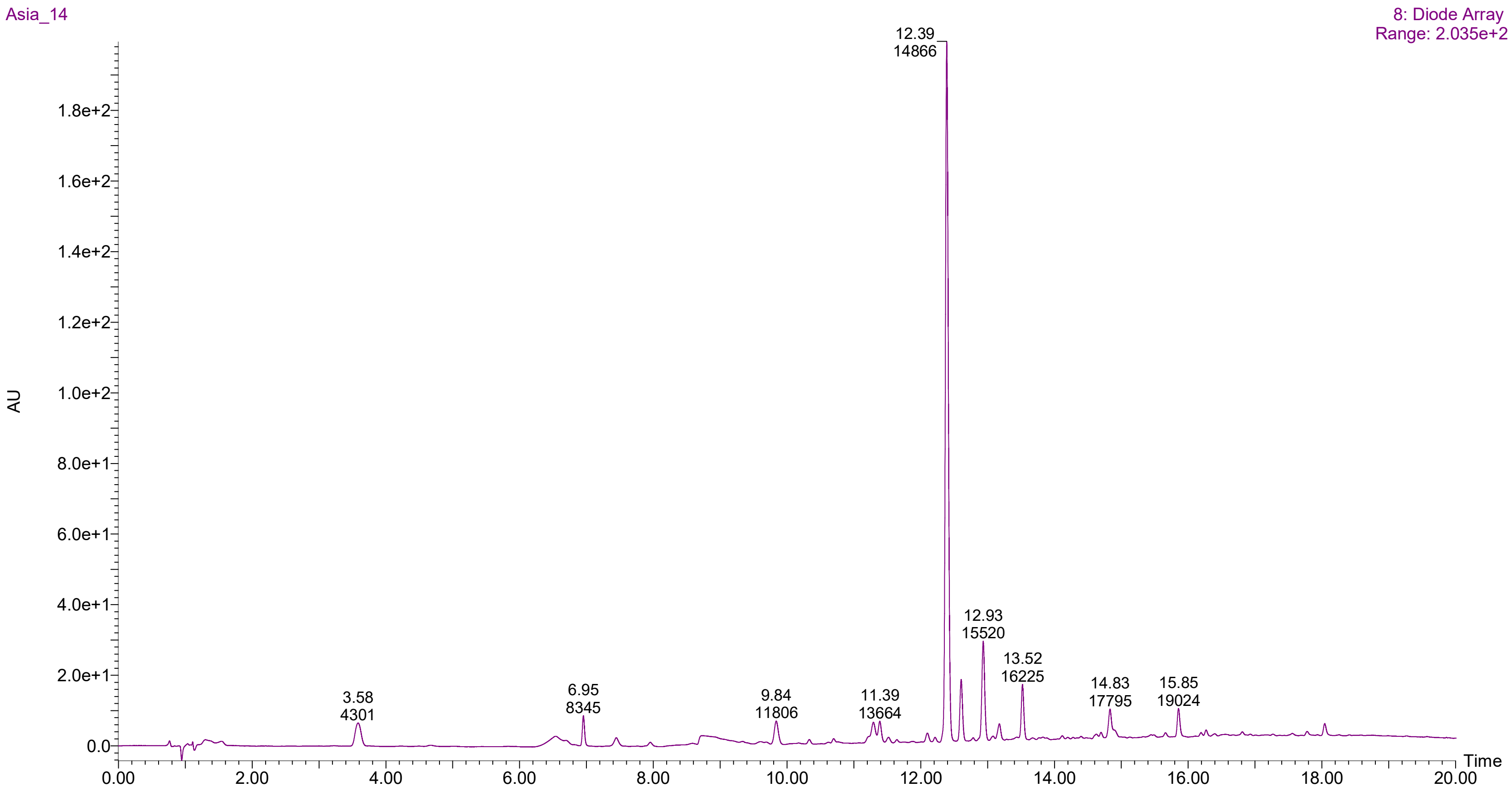

Figure 5. UPLC chromatogram of triticale bran

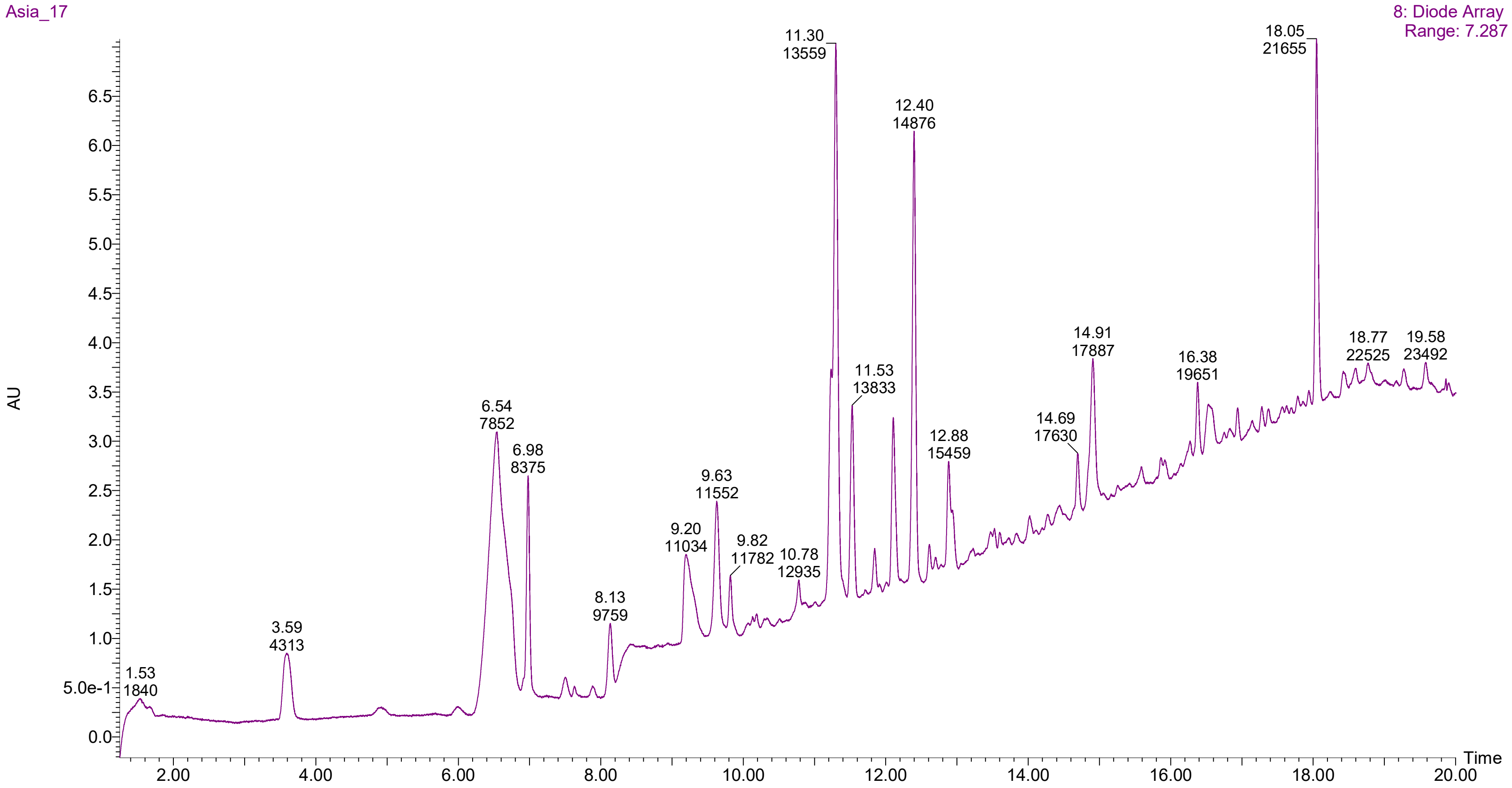

Figure 6. UPLC chromatogram of triticale bread
