# Supplementary material for: Content of Phenolic Acids in the Grain of Selected Polish Triticale Cultivars and Its Products
Source: Molecules. 2021 Jan 21;26(3):562. doi: 10.3390/molecules26030562 (PMC7865965; doi:10.3390/molecules26030562)
Supplement: Supplementary file 1 [file molecules-26-00562-s001.zip › Supplementary I.pdf]

1. 3-OH and 4-OH benzoic acids

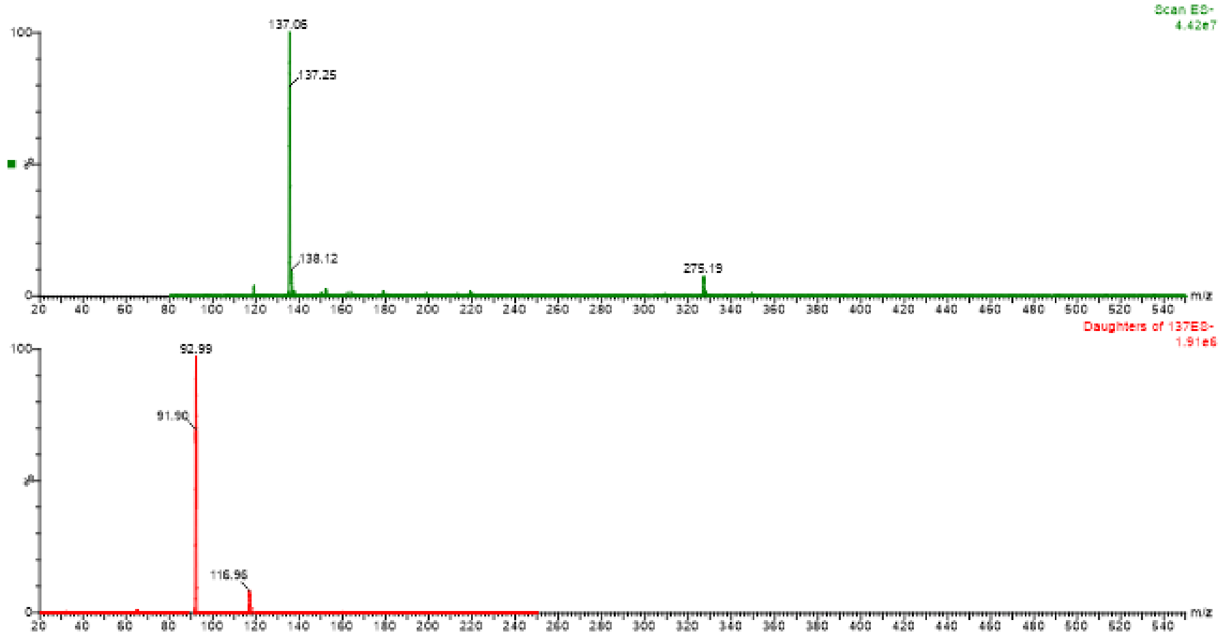

2. Caffeic acid

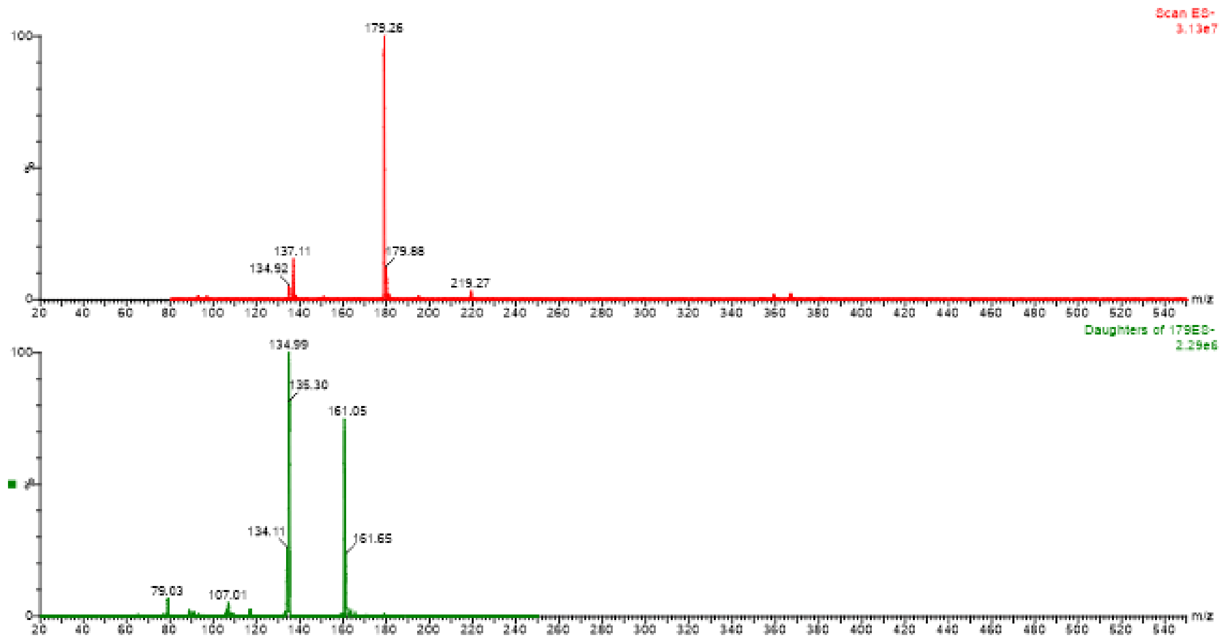

3. Syringic acid

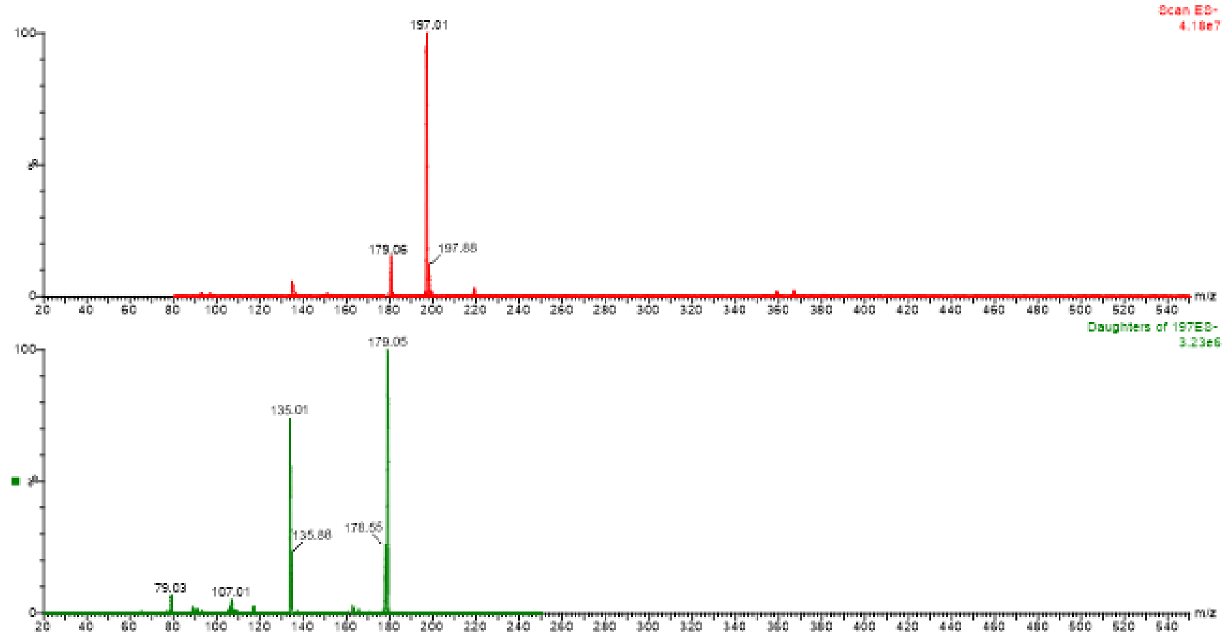

4. *p*-coumaric and *o*-coumaric acids

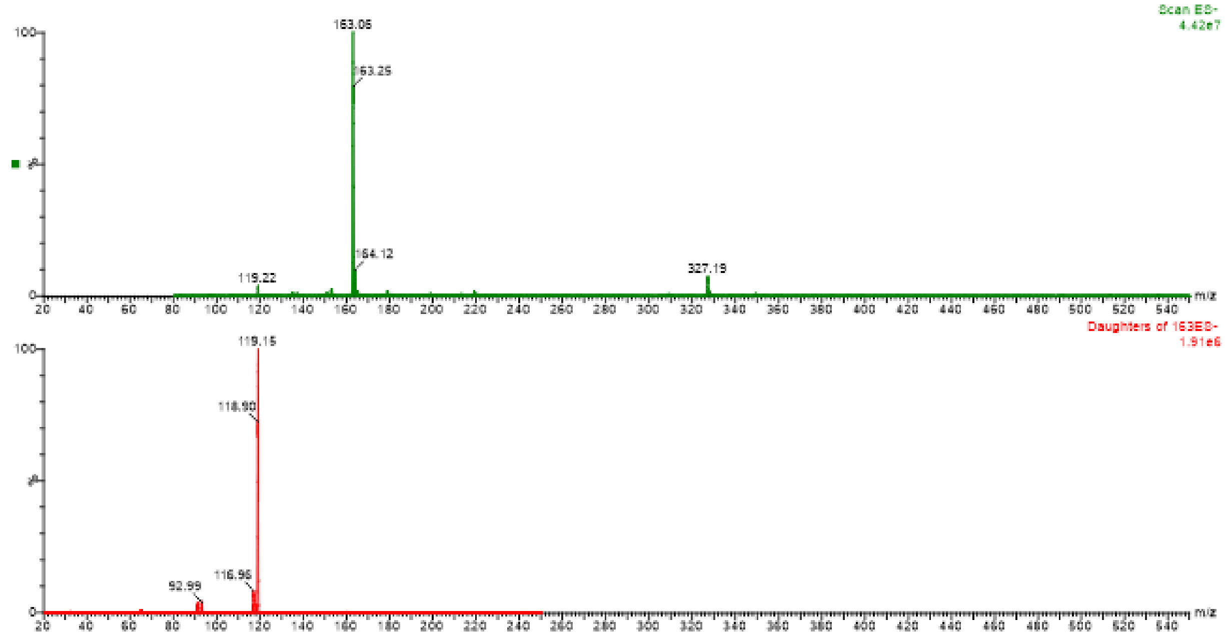

5. Ferulic acid

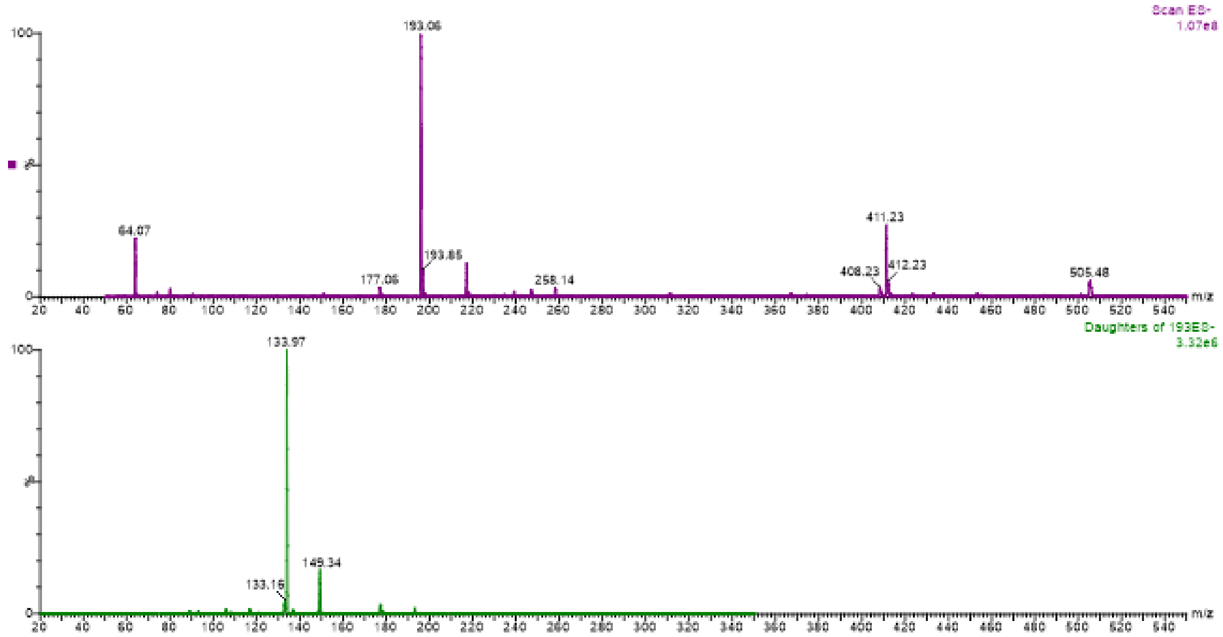

6. Sinapic acid

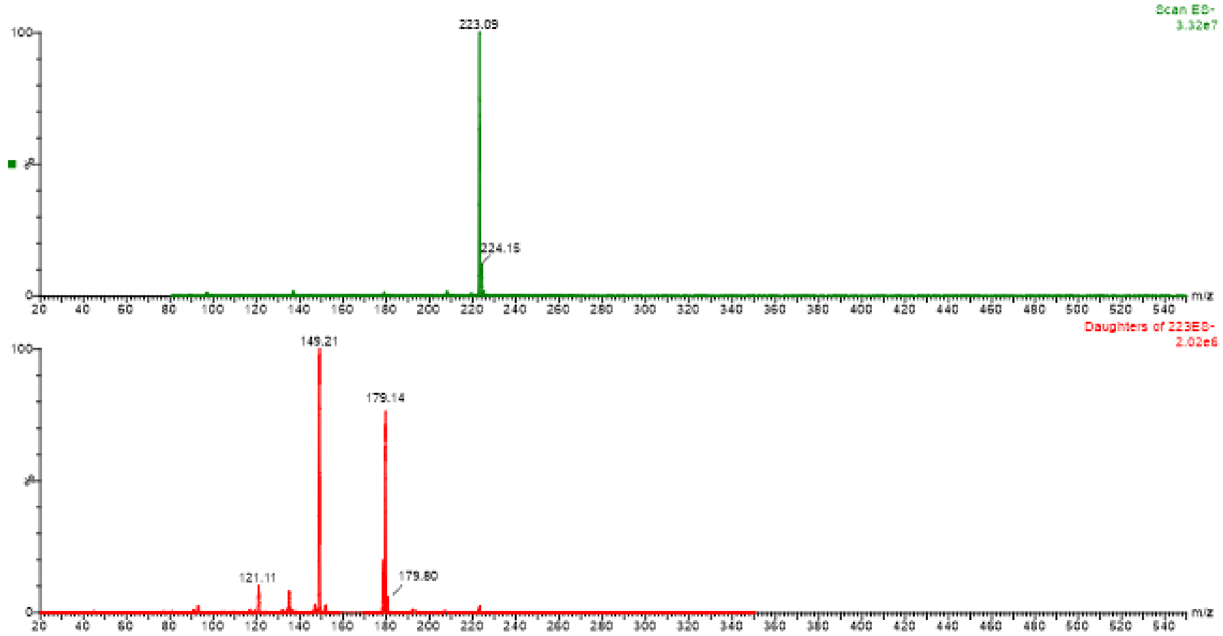

7. Di-ferulic acids

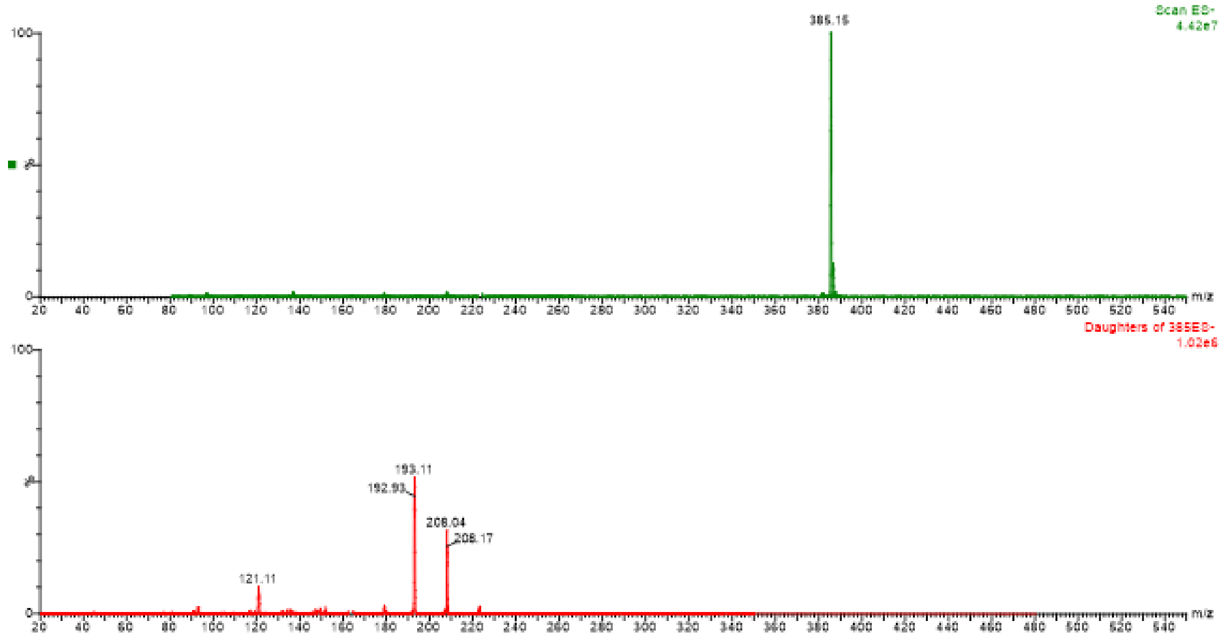

Figure 1. LC-MS/MS spectra of identified phenolic acids in triticale grain and its products.
Upper – pseudomolecular ion
Lower – daughter ions after MS/MS
